# Novel optimum contribution selection methods accounting for conflicting objectives in breeding programs for livestock breeds with historical migration

**DOI:** 10.1186/s12711-017-0320-7

**Published:** 2017-05-12

**Authors:** Yu Wang, Jörn Bennewitz, Robin Wellmann

**Affiliations:** Institute of Animal Science, University of Hohenheim, 70593 Stuttgart, Germany

## Abstract

**Background:**

Optimum contribution selection (OCS) is effective for increasing genetic gain, controlling the rate of inbreeding and enables maintenance of genetic diversity. However, this diversity may be caused by high migrant contributions (MC) in the population due to introgression of genetic material from other breeds, which can threaten the conservation of small local populations. Therefore, breeding objectives should not only focus on increasing genetic gains but also on maintaining genetic originality and diversity of native alleles. This study aimed at investigating whether OCS was improved by including MC and modified kinships that account for breed origin of alleles. Three objective functions were considered for minimizing kinship, minimizing MC and maximizing genetic gain in the offspring generation, and we investigated their effects on German Angler and Vorderwald cattle.

**Results:**

In most scenarios, the results were similar for Angler and Vorderwald cattle. A significant positive correlation between MC and estimated breeding values of the selection candidates was observed for both breeds, thus traditional OCS would increase MC. Optimization was performed under the condition that the rate of inbreeding did not exceed 1% and at least 30% of the maximum progress was achieved for all other criteria. Although traditional OCS provided the highest breeding values under restriction of classical kinship, the magnitude of MC in the progeny generation was not controlled. When MC were constrained or minimized, the kinship at native alleles increased compared to the reference scenario. Thus, in addition to constraining MC, constraining kinship at native alleles is required to ensure that native genetic diversity is maintained. When kinship at native alleles was constrained, the classical kinship was automatically lowered in most cases and more sires were selected. However, the average breeding value in the next generation was also lower than that obtained with traditional OCS.

**Conclusions:**

For local breeds with historical introgressions, current breeding programs should focus on increasing genetic gain and controlling inbreeding, as well as maintaining the genetic originality of the breeds and the diversity of native alleles via the inclusion of MC and kinship at native alleles in the OCS process.

**Electronic supplementary material:**

The online version of this article (doi:10.1186/s12711-017-0320-7) contains supplementary material, which is available to authorized users.

## Background

In recent decades, the widespread use of artificial insemination and other reproductive technologies has resulted in substantial genetic gains in livestock populations. However, another consequence is that only a limited number of animals with high estimated breeding values (EBV) have been intensively used in breeding programs, which can result in increasing rates of inbreeding to undesired levels. A high rate of inbreeding not only leads to considerable reduction in genetic variation but also more deleterious recessive alleles become homozygous, which may threaten the entire future of the population [1]. Thus, there is a conflict between maximizing genetic gain and managing the rate of inbreeding.

Crossbreeding has been demonstrated to be an efficient method to reduce the threat of inbreeding depression and increase the level of genetic diversity [2]. In addition, local breeds are often crossed with breeds of high economic value to improve performance. However, such introgressions of genetic material can be a threat for maintaining local breeds. Amador et al. [3] confirmed that, after several generations without management, even a small introduction of foreign genetic material will rapidly disperse throughout the original population, and that this material is difficult to remove. Therefore, foreign introgressions present a large risk for the conservation of local breeds, which leads to a conflict in current breeding programs between increasing the contribution of foreign genetic material and conserving local breeds.

Optimum contribution selection (OCS) is a selection method that is effective at achieving a balance between rate of inbreeding and genetic gain. This selection process maximizes genetic gain in the next generation while constraining the rate of inbreeding via restriction of relatedness among offspring [4–6]. The superiority of OCS has been demonstrated with both simulated [7, 8] and real data [9–11]. The objective function for OCS has been optimized using Lagrange multipliers [4, 8, 12], evolutionary algorithms [7, 13, 14], and semidefinite programming algorithms [9, 15, 16]. A similar related optimization problem was expressed as a mixed-integer quadratically constrained optimization problem and solved with branch-and-bound algorithms [17]. In this paper, we applied the algorithm described in [18] for solving cone-constrained convex problems by using R package *optiSel*.

OCS is efficient for controlling the level of kinship among progeny and the rate of inbreeding in future generations and can ultimately maintain genetic diversity [12, 16, 19, 20]. However, a high level of genetic diversity can be achieved by a large genetic contribution from migrant breeds, which is undesirable for the conservation of local breeds, because it reduces their genetic uniqueness, as well as the genetic diversity between breeds [21]. Thus, conflicting objectives are observed with regards to maintaining genetic diversity and conserving genetic uniqueness of local small breeds with historical migrations.

Instead of focusing on genetic gain and rate of inbreeding only, a reasonable breeding objective would be to also include recovery of genetic originality by reducing migrant contributions (MC). The diversity of native alleles may also be important for conservation. Thus, to conserve breeds with historical migrations, Wellmann et al. [22] recommended that approaches should not only constrain MC, but also aim at increasing the probability that alleles originating from native founders are not identical by descent (IBD).

Our aim was to investigate whether including MC and modified kinship matrices that account for breed origin of alleles as additional constraints in OCS can improve breeding programs in local breeds. Both conservation progress and genetic gain were evaluated. The following scenarios based on different objective functions were considered: (1) maximizing the diversity of native alleles while restricting MC and/or the average breeding value of the progeny generation at desired levels; (2) minimizing MC while restricting the loss of diversity of native alleles and/or the average breeding value of the progeny generation at desired levels; and (3) maximizing the average breeding value of the progeny generation while restricting MC and/or the loss of diversity of native alleles at desired levels. The traditional pedigree-based kinship was constrained in all optimization scenarios.

## Methods

### Data

Data from two local German cattle breeds, Angler and Vorderwald, were analyzed. The Angler breed is mainly located in the northern part of Germany and represents a dual-purpose breed, although the primary emphasis is on milk production. With the introduction of other breeds to improve milk yield, the Angler breed has experienced a considerable amount of migrant breed introgressions [23]. The Angler dataset was provided by the VIT (Vereinigte Informationssysteme Tierhaltung w.V., Verden), Germany. The Vorderwald breed is a dual-purpose breed located in the black forest region of southwest Germany. Similarly, due to their frequent crossing with high-yield breeds, the genetic originality of Vorderwald cattle has decreased dramatically [24, 25]. The Vorderwald dataset was provided by the Institute for Animal Breeding, Bavarian State Research Center for Agriculture in Grub, Germany. Both datasets consist of pedigrees with information on sex, breed, birth year and estimated breeding values for milk production obtained from routine genetic evaluations. Animals with an unknown pedigree born before 1970 were classified as purebred. Animals from other breeds and animals with an unknown pedigree born after 1970 were considered as migrants, although some may have purebred ancestors. The Angler dataset included 109,109 animals born between 1906 and 2015, of which 86,269 (79.1%) were classified as Angler. The Vorderwald dataset included 200,468 animals born between 1906 and 2010, of which 180,646 (90.1%) were classified as Vorderwald. MC for each animal was calculated and expressed as the proportion of migrant breed alleles based on pedigree information.

### Selection candidates

Selection candidates were chosen among animals that were classified as purebred in the herdbook in order to compute their optimum contributions with different approaches. Sires that had progeny born in 2005 and 2006 were set as male selection candidates and selected males were mated to 1000 randomly chosen dams, which are called female selection candidates. For the Angler breed, 1199 selection candidates were available and 15,370 animals were involved in the pedigree that included all selection candidates and their ancestors. For the Vorderwald breed, 1123 selection candidates were available and 12,934 animals were involved in the pedigree. For a better comparison of results between the two breeds, EBV were normalized across all selection candidates of each breed, with a mean of 0 and a standard deviation of 1.

### Optimum contribution selection strategies

The output of the optimum contribution selection procedure is a vector **c** with individual genetic contributions. The genetic contribution ${\text{c}}_{i}$ of animal $i$ is the fraction of genes in the next generation that originate from this individual. Genetic contributions cannot be negative, i.e. ${\text{c}}_{\text{i}} \ge 0$, which is denoted as constraint (a) in the following. The total genetic contribution of each sex must be equal to 0.5 for diploid species, i.e. ${\mathbf{c}}^{{\mathbf{\prime }}} {\mathbf{s}} = 0.5$ and ${\mathbf{c}}^{{\mathbf{\prime }}} {\mathbf{d}} = 0.5$ (constraint b), where ${\mathbf{s}}$ and ${\mathbf{d}}$ are vectors of the indicators (0/1) of a candidate’s sex. Because cows can produce only a limited number of calves, all female selection candidates were used for breeding and the genetic contributions were forced to be equal, i.e. ${\text{c}}_{{{\text{d}}_{1} }} = {\text{c}}_{{{\text{d}}_{2} }} = \cdots = {\text{c}}_{{{\text{d}}_{\text{n}} }}$ (constraint c). Thus, optimization was only performed for bulls. For male selection candidates, the number of offspring is not limited, thus the maximum genetic contribution is 0.5, i.e. ${\text{c}}_{{{\text{s}}_{\text{i}}  }} \le 0.5$. To calculate the proportion of sires with non-zero genetic contributions, a sire $i$ is considered to have a non-zero genetic contribution only if ${\text{c}}_{{s_{i} }} \ge 0.00025$ to account for possible numerical inaccuracies of the algorithm.

Four kinships that are involved in the calculation of the OCS procedure were applied. The diversity parameters described in [22] are complementary to the kinships used here, i.e. these kinship values are equal to 1 minus the corresponding diversity denoted as $\varphi_{A} , \ldots , \varphi_{D}$ in [22]. The relevant derivations of the formulas for calculating the diversity parameters are provided in detail in [22].

The classic kinship ${\text{f}}_{\text{A}}$ between individuals $i$ and $j$ (element of matrix ${\mathbf{f}}_{{\mathbf{A}}}$), which describes the probability that two alleles, $X_{i}$ and $X_{j}$, at a locus that are randomly selected from individuals $i$ and $j$ are IBD (i.e. ), was restricted in all scenarios. For breeds with historical migrations and foreign introgressions, Wellmann et al. [22] proposed that the breed origin of the alleles should be considered to preserve the local breed. Thus, we considered different approaches that account for the origin of alleles, denoted as $\varvec{ }{\text{f}}_{\text{B}}$, ${\text{f}}_{\text{C}}$ and ${\text{f}}_{\text{D}}$. Kinship matrix ${\mathbf{f}}_{{\mathbf{B}}}$ contains the probabilities that two alleles randomly chosen from two individuals at a locus are IBD or that at least one allele is from a migrant breed (${\varvec{\mathcal{M}}}$):
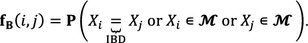


Note that this is equal to the probability that both alleles are IBD and native plus the probability that at least one allele is from a migrant.

Kinship matrix ${\mathbf{f}}_{{\mathbf{C}}}$ contains the probabilities that two alleles randomly chosen from two individuals at a locus are IBD or both alleles are from migrant breeds:
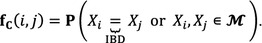


This is equal to ${\mathbf{f}}_{{\mathbf{B}}} \left( {i,j} \right) = {\mathbf{f}}_{{\mathbf{C}}} \left( {i,j} \right) + \varvec{P}\left( {either\varvec{ }X_{i} \in {\varvec{\mathcal{M}}}\;{\mathbf{ }}{\text{or}}\; X_{j} \in {\varvec{\mathcal{M}}}} \right)$. The probability that at least one of the two randomly chosen alleles is from a migrant breed is higher than the probability that both are from migrant breeds. Thus, ${\mathbf{f}}_{{\mathbf{B}}}$ is greater than ${\mathbf{f}}_{{\mathbf{C}}}$. In general, ${\mathbf{f}}_{{\mathbf{A}}} \le {\mathbf{f}}_{{\mathbf{C}}} \le {\mathbf{f}}_{{\mathbf{B}}}$ (element-wise). The kinship at native alleles ${\text{f}}_{\text{D}}$ is defined as the conditional probability that two alleles *X* and *Y* at a locus that are randomly chosen from the offspring population are IBD, given that both descended from native founders (${\varvec{\mathcal{F}}}$):
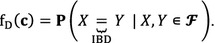


Note that this value says nothing about the kinship at loci that originate from migrants or about the MC. The mean kinships for the offspring generation are ${\mathbf{c^{\prime}f}}_{{\mathbf{A}}} {\mathbf{c}}$, ${\mathbf{c^{\prime}f}}_{{\mathbf{B}}} {\mathbf{c}}$ and ${\mathbf{c^{\prime}f}}_{{\mathbf{C}}} {\mathbf{c}}$, respectively. Mean kinship ${\text{f}}_{\text{D}}$ in the offspring population was calculated as ${\text{f}}_{\text{D}} \left( {\mathbf{c}} \right) = 1 - \frac{{1 - {\mathbf{c}}^{{\mathbf{\prime }}} {\mathbf{f}}_{{\mathbf{B}}} {\mathbf{c}}}}{{{\mathbf{c}}^{{\mathbf{\prime }}} {\mathbf{f}}_{{\mathbf{N}}} {\mathbf{c}}}}$, where ${\mathbf{f}}_{{\mathbf{N}}}$ is a matrix containing the probabilities that both randomly chosen alleles at a locus originated from native founders.

Our aim was to identify the best method of accounting for the conflicting objectives of a breeding program, which are to increase breeding values, to maintain genetic diversity, and to maintain genetic originality of the breed. Since $1 - {\text{f}}_{\text{D}} ({\mathbf{c}})={\mathbf{P}}({X{\mathop{\ne}\limits_{{{\mathbf{IBD}}}}}Y |X,Y \in {\varvec{\mathcal{F}}}})$ is the genetic diversity at native alleles, the constraint on ${\text{f}}_{\text{D}}$ is used to maintain or increase genetic diversity at native alleles and is a parameter of interest. Kinship ${\text{f}}_{\text{B}}$ and ${\text{f}}_{\text{C}}$ were considered because minimizing or constraining ${\text{f}}_{\text{D}}$ is in general not a convex problem, so minimizing ${\text{f}}_{\text{B}}$ and ${\text{f}}_{\text{C}}$ could result in lower ${\text{f}}_{\text{D}}$ values than minimizing ${\text{f}}_{\text{D}}$ itself.

In the different scenarios, an upper bound for MC (${\text{ub}}.{\text{MC}}$) and/or a lower bound for the average EBV (${\text{lb}}.{\text{EBV}}$) were set as additional constraints. The expectation of the average EBV in the next generation is ${\mathbf{c}}^{{\mathbf{\prime }}} {\mathbf{EBV}}$, where ${\mathbf{EBV}}$ is a vector of the EBV of each selection candidate. The expectation of the average MC of the next generation is ${\mathbf{c}}^{{\mathbf{\prime }}} {\mathbf{MC}}$, where ${\mathbf{MC}}$ is a vector of the MC of each selection candidate.

For all optimization problems, constraints a, b, and c were applied to limit the solution for ${\text{c}}_{\text{i}}$ to within a reasonable range. Solver “cccp” [18], which was called from the R package optiSel [26], was used to solve the optimization problems. This solver contains routines for solving cone constrained convex problems using interior-point methods that are partially ported from Python’s CVXOPT and based on Nesterov-Todd scaling [27]. The solver uses a primal–dual path following algorithms for linear and quadratic cone constrained programming.

Scenarios were categorized based on three main objective functions: minimizing kinships, minimizing MC and maximizing genetic gain in the next generation. For minimizing kinships, three sub-scenarios were considered, which involved minimizing ${\text{f}}_{\text{B}}$, ${\text{f}}_{\text{C}}$ and ${\text{f}}_{\text{D}}$, respectively. Parameters ${\text{ub}}.{\text{f}}_{\text{A}}$, ${\text{ub}}.{\text{f}}_{\text{B}}$,${\text{ub}}.{\text{f}}_{\text{C}}$, ${\text{ub}}.{\text{f}}_{\text{D}}$ and ${\text{ub}}.{\text{MC}}$ were defined as the upper bound values of the corresponding parameters in the next generation, whereas ${\text{lb}}.{\text{EBV}}$ was set as the lower bound of the mean EBV for the next generation. One or several of the following constraints were used to define the optimization problems for each breed:

$$ {\mathbf{c^{\prime}f}}_{{\mathbf{A}}} {\mathbf{c}} \le {\text{ub}}.{\text{f}}_{\text{A}} , $$

$$ {\mathbf{c^{\prime}f}}_{{\mathbf{B}}} {\mathbf{c}} \le {\text{ub}}.{\text{f}}_{\text{B}} , $$

$$ {\mathbf{c^{\prime}f}}_{{\mathbf{C}}} {\mathbf{c}} \le {\text{ub}}.{\text{f}}_{\text{C}} , $$

$$ {\text{f}}_{\text{D}} \left( {\mathbf{c}} \right) \le {\text{ub}}.{\text{f}}_{\text{D}} , $$

$$ {\mathbf{c^{\prime}MC}} \le {\text{ub}}.{\text{MC,}} $$

$$ {\mathbf{c^{\prime}EBV}} \ge {\text{lb}}.{\text{EBV}} . $$

The OCS scenarios considered are listed in Table 1. The name of each optimization scenario consists of a prefix that indicates the objective function and a suffix that indicates the constraint settings. For example, scenario *maxEBV.A.B.MC* indicates a scenario that maximizes the average EBV in the next generation, while constraining ${\text{f}}_{\text{A}}$, ${\text{f}}_{\text{B}}$, and MC. The vector of genetic contributions for this scenario is denoted as ${\mathbf{c}}_{{   {\text{maxEBV}}.{\text{A}}.{\text{B}}.{\text{MC}}}}$.

**Table 1:** Names of the OCS scenarios based on different objective functions

Objective function	Name of the scenario^a^
Minimizing ${\text{f}}_{\text{B}}$	*minfB.A; minfB.A.MC; minfB.A.MC.EBV*
Minimizing ${\text{f}}_{\text{C}}$	*minfC.A; minfC.A.MC; minfC.A.MC.EBV*
Minimizing ${\text{f}}_{\text{D}}$	*minfD.A; minfD.A.MC; minfD.A.MC.EBV*
Minimizing MC	*minMC.A; minMC.A.EBV; minMC.A.B.EBV; minMC.A.C.EBV; minMC.A.D.EBV*
Maximizing EBV	*maxEBV.A; maxEBV.A.MC; maxEBV.A.B.MC; maxEBV.A.C.MC; maxEBV.A.D.MC*

^a^The name of each optimization scenario consists of a prefix that indicates the objective function and a suffix that indicates the constraint settings. For example, scenario *minfB.A* indicates that the objective function is to minimize the average ${\text{f}}_{\text{B}}$ value in the following generation with a constraint on ${\text{f}}_{\text{A}}$

Criteria for comparing scenarios included not only the result of the objective function, but also the other parameters obtained in the scenario, in particular EBV, MC, classic kinship, and kinship at native alleles. To evaluate the effectiveness of the OCS scenarios, the results were compared with the output from a reference scenario (*REF*) and the output from a truncation selection scenario (*TS*). In scenario *REF* all selection candidates were used as parents and had equal contributions to the offspring generation. For endangered breeds, an effective population size (${\text{N}}_{\text{e}}$) of 50 is often considered as sufficient [28]. Based on the equation in [1], $\frac{1}{{{\text{N}}_{\text{e}} }} = \frac{1}{{4*{\text{N}}_{\text{sire}} }} + \frac{1}{{4*{\text{N}}_{\text{dam}} }}$, the 13 sires with the highest EBV were selected as male selection candidates in the *TS* scenario, and mated to the 1000 dams. All parents had equal contributions to the offspring generation in this scenario.

To ensure that optimal solutions exist in all scenarios for each breed, feasible threshold values must be set for the constraints. To restrict the rate of inbreeding, the upper bound (${\text{ub}}.{\text{f}}_{\text{A}}$) was defined as follows. When ${\text{N}}_{\text{e}}$ is equal to 50, the rate of inbreeding $\Delta {\text{F}}$, which can be calculated from $\Delta {\text{F}} = \frac{1}{{2{\text{N}}_{\text{e}} }}$, is 1% per generation. Based on this, the threshold for ${\text{f}}_{\text{A}}$ was calculated as ${\text{ub}}.{\text{f}}_{\text{A}} = \overline{{{\text{f}}_{\text{A}} }} + \left( {1 - \overline{{{\text{f}}_{\text{A}} }} } \right)  \Delta {\text{F}}$, where $\overline{{{\text{f}}_{\text{A}} }}$ is the average kinship of the selection candidates.

To calculate the constraint setting for the other parameters, we used the results from the scenario that optimizes the corresponding parameter with restriction only on ${\text{f}}_{\text{A}}$ and the *REF* scenario, using the following calculations:

$$ {\text{ub}}.{\text{f}}_{\text{B}} =\uplambda{\mathbf{c}}_{{{\text{minfB}}.{\text{A}}}}^{\prime } {\mathbf{f}}_{{\mathbf{B}}} {\mathbf{c}}_{{{\text{minfB}}.{\text{A}}}} + \left( {1 -\uplambda} \right){\mathbf{c}}_{\text{REF}}^{\prime } {\mathbf{f}}_{{\mathbf{B}}} {\mathbf{c}}_{\text{REF}} , $$

$$ {\text{ub}}.{\text{f}}_{\text{C}} =\uplambda{\mathbf{c}}_{{{\text{minfC}}.{\text{A}}}}^{\prime } {\mathbf{f}}_{{\mathbf{C}}} {\mathbf{c}}_{{{\text{minfC}}.{\text{A}}}} + \left( {1 -\uplambda} \right){\mathbf{c}}_{\text{REF}}^{\prime } {\mathbf{f}}_{{\mathbf{C}}} {\mathbf{c}}_{\text{REF}} , $$

$$ {\text{ub}}.{\text{f}}_{\text{D}} =\uplambda{\mathbf{f}}_{{\mathbf{D}}} \left( {{\mathbf{c}}_{{{\text{minfD}}.{\text{A}}}} } \right) + \left( {1 -\uplambda} \right){\mathbf{f}}_{{\mathbf{D}}} \left( {{\mathbf{c}}_{\text{REF}} } \right), $$

$$ {\text{ub}}.{\text{MC}} =\uplambda{\mathbf{c}}_{{{\text{minMC}}.{\text{A}}}}^{\prime } {\mathbf{MC}} + \left( {1 -\uplambda} \right){\mathbf{c}}_{\text{REF}}^{\prime } {\mathbf{MC}}, $$

$$ {\text{lb}}.{\text{EBV}} =\uplambda{\mathbf{c}}_{{{\text{maxEBV}}.{\text{A}}}}^{\prime } {\mathbf{EBV}} + \left( {1 -\uplambda} \right){\mathbf{c}}_{\text{REF}}^{\prime } {\mathbf{EBV}}, $$

where $\uplambda$ is a parameter that indicates the proportion of progress to be accomplished for each constrained parameter relative to the scenario with a restriction only on ${\text{f}}_{\text{A}}$. The value of $\uplambda$ can be determined by the breeding organization. A higher $\uplambda$ value indicates a stricter setting for all constraints. We set $\uplambda$ at 0.3 to ensure that optimized solutions were found for all scenarios and for both breeds. The specific values used for all constraints for each breed are in Additional file 1: Table S1.

## Results

**Fig. 1:**
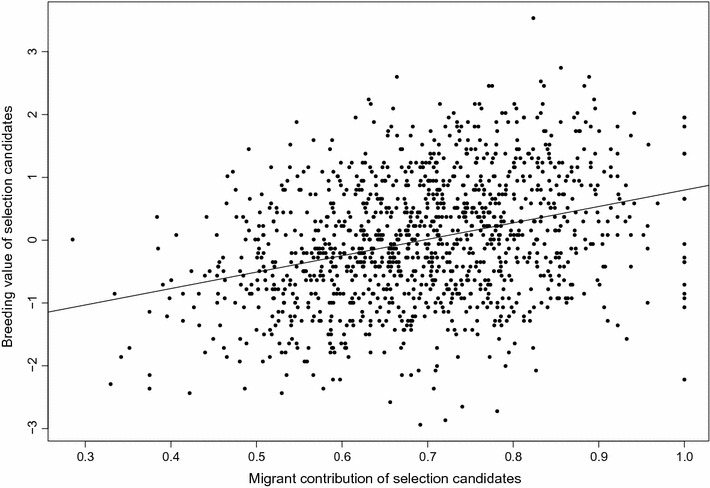
Relationship between migrant contribution and the estimated breeding value of selection candidates in the Angler cattle population. The correlation between the EBV and MC is 0.328 and the regression coefficient between the EBV and MC is 2.614

**Fig. 2:**
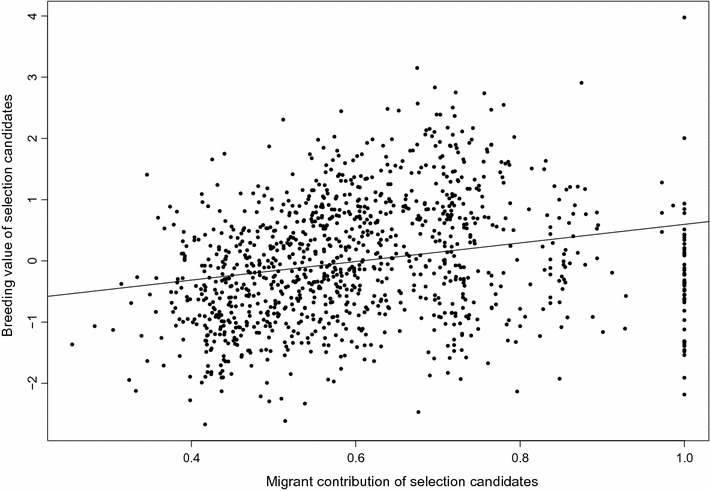
Relationship between migrant contribution and the estimated breeding value of selection candidates in the Vorderwald cattle population. The correlation between the EBV and MC is 0.232 and the regression coefficient between the EBV and MC is 1.517

Results of the basic statistical analyses for average kinship, MC and EBV of the parent generation are in Table 2 for both breeds. Average kinship ${\text{f}}_{\text{A}}$ was lower for the Angler population than for the Vorderwald population (0.020 vs. 0.025) but ${\text{f}}_{\text{B}}$ (0.910 vs. 0.853) and ${\text{f}}_{\text{C}}$ levels (0.488 vs. 0.381) were higher. On average, 69.5 and 60.7% of the genetic material of the Angler and Vorderwald cattle, respectively, originated from migrant breeds. Native effective population sizes of 86 and 49 were estimated from six previous generations for Angler and Vorderwald cattle, respectively. Native effective population size is a parameter that quantifies the decrease in native allele diversity and is defined in [22]. If the native effective size is high, then native allele diversity decreases slowly. Thus, the diversity of native alleles decreased more rapidly in Vorderwald cattle than in Angler cattle, whereas MC were higher in Angler cattle. Average EBV for both breeds were below the current population mean, which is 100 for Angler and 0 for Vorderwald because selection candidates were sampled from old age cohorts. A positive correlation between EBV and MC was found for both breeds (Figs. 1, 2).

**Table 2:** Descriptive statistics for the active breeding population in the Angler and Vorderwald breeds

	Angler (N = 1199)		Vorderwald (N = 1123)	
	Mean	SD	Mean	SD
${\text{f}}_{\text{A}}$	0.020	0.027	0.025	0.027
${\text{f}}_{\text{B}}$	0.910	0.055	0.853	0.084
${\text{f}}_{\text{C}}$	0.488	0.123	0.381	0.128
MC	0.695	0.126	0.607	0.153
EBV	86.868	13.901	−512.020	502.465

### Minimizing average kinship

Genetic contributions of the selection candidates were optimized to minimize ${\text{f}}_{\text{B}} ,$ ${\text{f}}_{\text{C}}$ and ${\text{f}}_{\text{D}}$ with restrictions on MC and/or average EBV in the offspring generation for each breed, (see Tables 3, 4, 5, respectively). Compared to the *REF* scenario, all OCS scenarios showed superior results for the optimized criteria as expected.

**Table 3:** Optimization of the genetic contributions when minimizing kinship $\varvec{f}_{\varvec{B}}$ with a restriction on migrant contribution and/or mean estimated breeding values

Scenario^a^	Parameter^b^							
	${\mathbf{f}}_{{\mathbf{A}}}$	${\mathbf{f}}_{{\mathbf{B}}}$ ^c^	${\mathbf{f}}_{{\mathbf{C}}}$	${\mathbf{f}}_{{\mathbf{D}}}$	MC	EBV	Selected^d^	SD of ${\mathbf{c}}_{{\mathbf{s}}}$ ^e^
Angler								
*REF*	0.022	0.926	0.527	0.049	0.722	0.211	–	–
*TS*	0.031	0.939	0.565	0.067	0.722	1.184	0.065	0
*minfB.A*	*0.030*	0.827	0.345	0.082	0.570	−0.295	0.106	0.012
*minfB.A.MC*	*0.030*	0.827	0.345	0.082	***0.570***	−0.295	0.111	0.012
*minfB.A.MC.EBV*	*0.030*	0.866	0.404	0.083	***0.623***	*0.516*	0.081	0.012
Vorderwald								
*REF*	0.030	0.852	0.380	0.072	0.605	0.287	–	–
*TS*	0.043	0.882	0.432	0.093	0.645	1.161	0.106	0
*minfB.A*	*0.035*	0.789	0.300	0.074	0.528	−0.111	0.260	0.011
*minfB.A.MC*	*0.035*	0.789	0.300	0.074	***0.528***	−0.111	0.260	0.011
*minfB.A.MC.EBV*	*0.035*	0.813	0.327	0.075	***0.555***	*0.550*	0.228	0.010

^a^The name of each optimization scenario consists of a prefix that indicates the objective function and a suffix that indicates the constraint settings

^b^The parameter used as a constraint is marked in italics in the scenario. Bold italic values indicate that the actual value obtained does not reach the limit of the corresponding constraint (value higher than the lower limit or lower than the upper limit)

^c^Objective function

^d^Proportion of selected sires with non-zero genetic contributions; a ${\text{c}}_{{{\text{s}}_{\text{i}} }}$ value lower than 0.00025 is treated as zero

^e^Standard deviation of the genetic contributions of all male selection candidates

**Table 4:** Optimization of the genetic contribution when minimizing kinship $\varvec{f}_{\varvec{C}}$ with a restriction on migrant contribution and/or mean estimated breeding values

Scenario^a^	Parameter^b^							
	${\mathbf{f}}_{{\mathbf{A}}}$	${\mathbf{f}}_{{\mathbf{B}}}$	${\mathbf{f}}_{{\mathbf{C}}}$ ^c^	${\mathbf{f}}_{{\mathbf{D}}}$	MC	EBV	Selected^d^	SD of ${\mathbf{c}}_{{\mathbf{s}}}$ ^e^
Angler								
*REF*	0.022	0.926	0.527	0.049	0.722	0.211	–	–
*TS*	0.031	0.939	0.565	0.067	0.722	1.184	0.065	0
*minfC.A*	*0.030*	0.827	0.345	0.082	0.570	−0.299	0.111	0.012
*minfC.A.MC*	*0.030*	0.827	0.345	0.082	***0.570***	−0.299	0.111	0.012
*minfC.A.MC.EBV*	*0.030*	0.866	0.404	0.083	***0.623***	*0.516*	0.091	0.012
Vorderwald								
*REF*	0.030	0.852	0.380	0.072	0.605	0.287	–	–
*TS*	0.043	0.882	0.432	0.093	0.645	1.161	0.106	0
*minfC.A*	*0.035*	0.789	0.300	0.074	0.528	−0.109	0.276	0.010
*minfC.A.MC*	*0.035*	0.789	0.300	0.074	***0.528***	−0.109	0.276	0.010
*minfC.A.MC.EBV*	*0.035*	0.813	0.327	0.075	***0.555***	*0.550*	0.228	0.010

^a^The name of each optimization scenario consists of a prefix indicating the objective function and a suffix indicating the constraint settings

^b^The parameter used as a constraint is marked in italic in the scenario. Bold italic values show that the actual value obtained does not reach the limit of the corresponding constraint in this scenario (value higher than the lower limit or lower than the upper limit)

^c^Objective function

^d^Proportion of selected sires with non-zero genetic contributions; a ${\text{c}}_{{{\text{s}}_{\text{i}} }}$ value lower than 0.00025 is treated as zero

^e^Standard deviation of the genetic contributions of all male selection candidates

**Table 5:** Optimization of genetic contribution when minimizing kinship $\varvec{f}_{\varvec{D}}$ with restriction on migrant contribution and/or mean estimated breeding values

Scenario^a^	Parameter^b^							
	${\mathbf{f}}_{{\mathbf{A}}}$	${\mathbf{f}}_{{\mathbf{B}}}$	${\mathbf{f}}_{{\mathbf{C}}}$	${\mathbf{f}}_{{\mathbf{D}}}$ ^c^	MC	EBV	Selected^d^	SD of ${\mathbf{c}}_{{\mathbf{s}}}$ ^e^
Angler								
*REF*	0.022	0.926	0.527	0.049	0.722	0.211	–	–
*TS*	0.031	0.939	0.565	0.067	0.722	1.184	0.065	0
*minfD.A*	***0.020***	0.954	0.614	0.040	0.782	0.078	0.434	0.009
*minfD.A.MC*	***0.019***	0.899	0.464	0.044	*0.677*	0.090	0.414	0.004
*minfD.A.MC.EBV*	***0.020***	0.899	0.464	0.047	*0.677*	*0.516*	0.333	0.005
Vorderwald								
*REF*	0.030	0.852	0.380	0.072	0.605	0.287	–	–
*TS*	0.043	0.882	0.432	0.093	0.645	1.161	0.106	0
*minfD.A*	***0.035***	0.895	0.456	0.057	0.669	0.759	0.398	0.015
*minfD.A.MC*	***0.027***	0.833	0.352	0.058	*0.582*	0.145	0.472	0.006
*minfD.A.MC.EBV*	***0.029***	0.833	0.353	0.064	*0.582*	*0.550*	0.358	0.007

^a^The name of each optimization scenario consists of a prefix indicating the objective function and a suffix indicating the constraint settings

^b^The parameter used as a constraint is marked in italic in the scenario. Bold italic values show that the actual value obtained does not reach the limit of the corresponding constraint in this scenario (value higher than the lower limit or lower than the upper limit)

^c^Objective function

^d^Proportion of selected sires with non-zero genetic contributions; a ${\text{c}}_{{{\text{s}}_{\text{i}} }}$ value lower than 0.00025 is treated as zero

^e^Standard deviation of the genetic contributions of all male selection candidates

Table 3 shows the results obtained when minimizing ${\text{f}}_{\text{B}}$ in the offspring generation under the different constraints for each breed. The lowest ${\text{f}}_{\text{B}}$ for Angler cattle was 0.827 when the upper bound for ${\text{f}}_{\text{A}}$ in the next generation was set to 0.030. MC was lower than the constraint value setting (0.570 vs. 0.677). Thus, the minimum ${\text{f}}_{\text{B}}$ did not change after adding the constraint on MC (*minfB.A.MC*). When the restriction on average EBV was set to 0.516, the average kinship ${\text{f}}_{\text{B}}$ increased to 0.866, which was still lower than the ${\text{f}}_{\text{B}}$ obtained in the *REF* scenario (0.926). Similar results were obtained for Vorderwald cattle. When the upper bound for ${\text{f}}_{\text{A}}$ in the progeny generation was set to 0.035, the minimum ${\text{f}}_{\text{B}}$ level in the progeny generation was 0.789. Again, ${\text{f}}_{\text{B}}$ did not change after adding an upper bound for MC (0.528 vs. 0.582). ${\text{f}}_{\text{B}}$ increased to 0.813 when the EBV constraint was set to 0.550, although it was lower than the ${\text{f}}_{\text{B}}$ obtained in the *REF* scenario (0.852).

Results when minimizing ${\text{f}}_{\text{C}}$ were similar to minimizing ${\text{f}}_{\text{B}}$ (see Table 4). The ${\text{f}}_{\text{C}}$ of the progeny generation decreased to 0.345 for Angler cattle when the upper bound for ${\text{f}}_{\text{A}}$ was set to 0.030. When ${\text{f}}_{\text{C}}$ was minimized, MC decreased to a value lower than the constraint level setting (0.570 vs. 0.677). Thus, minimizing ${\text{f}}_{\text{C}}$ gave the same results for scenarios *minfC.A* and *minfC.A.MC*. After adding an EBV constraint of 0.516, ${\text{f}}_{\text{C}}$ increased to 0.404 but was lower than the ${\text{f}}_{\text{C}}$ obtained in the *REF* scenario (0.527). For Vorderwald cattle, the minimum average ${\text{f}}_{\text{C}}$ in the progeny generation was 0.300 when ${\text{f}}_{\text{A}}$ was restricted to 0.035, even after adding a higher constraint on MC (0.582 vs. 0.528). In scenario *minfC.A.MC.EBV*, ${\text{f}}_{\text{C}}$ reached 0.327 after adding an EBV constraint of 0.550, although this was lower than the ${\text{f}}_{\text{C}}$ obtained in the *REF* scenario (0.380).

When the kinship at native alleles, ${\text{f}}_{\text{D}}$, was minimized, the average kinship ${\text{f}}_{\text{A}}$ was automatically lowered in most cases (Table 5); in Angler cattle, ${\text{f}}_{\text{A}}$ reached 0.020, which was lower than the constraint level (0.030). In this case, the minimum ${\text{f}}_{\text{D}}$ was 0.040. When MC was restricted to 0.677, the minimum ${\text{f}}_{\text{D}}$ increased to 0.044. When an EBV constraint of 0.516 was added, the minimum ${\text{f}}_{\text{D}}$ increased to 0.047, which was still lower than the ${\text{f}}_{\text{D}}$ obtained in the *REF* scenario (0.049). For Vorderwald cattle, when ${\text{f}}_{\text{A}}$ was restricted to 0.035 in the progeny generation, the lowest ${\text{f}}_{\text{D}}$ was 0.057. When the maximum MC was set to 0.582, ${\text{f}}_{\text{D}}$ increased to 0.058. When adding an EBV constraint of 0.550, the lowest ${\text{f}}_{\text{D}}$ was 0.064, which was still lower than the ${\text{f}}_{\text{D}}$ obtained in the *REF* scenario (0.072).

### Minimizing migrant contribution

Table 6 shows the results of minimizing MC under various constraints. When ${\text{f}}_{\text{A}}$ was restricted to 0.030 in the progeny generation for Angler cattle, MC was equal to 0.570. When constraining the EBV to at least 0.516, MC in scenario *minMC.A.EBV* increased to 0.622 and ${\text{f}}_{\text{B}}$ and ${\text{f}}_{\text{C}}$ were lower than their constraint settings (0.866 vs. 0.896 and 0.404 vs. 0.472, respectively). Thus, adding constraints for ${\text{f}}_{\text{B}}$ or ${\text{f}}_{\text{C}}$ did not change the results. When the upper bound for ${\text{f}}_{\text{D}}$ was set to 0.046, MC increased to 0.683, which was less than that achieved in the *REF* scenario (0.722). Results were similar for Vorderwald cattle. The minimum MC achieved in the next generation was 0.527 when the upper bound for ${\text{f}}_{\text{A}}$ was 0.035. When the lower bound for EBV was set to 0.550, the minimal MC increased to 0.555. Adding a lower constraint for ${\text{f}}_{\text{B}}$ (0.813 vs. 0.833) or ${\text{f}}_{\text{C}}$ (0.327 vs. 0.356) did not change results. When the upper bound for ${\text{f}}_{\text{D}}$ was set to 0.067 as an additional constraint, the minimum MC was 0.571, which was less than that obtained in the *REF* scenario (0.605).

**Table 6:** Optimization of the genetic contribution when minimizing the migrant contribution with restricted kinship and/or mean estimated breeding values

Scenario^a^	Parameter^b^							
	${\mathbf{f}}_{{\mathbf{A}}}$	${\mathbf{f}}_{{\mathbf{B}}}$	${\mathbf{f}}_{{\mathbf{C}}}$	${\mathbf{f}}_{{\mathbf{D}}}$	MC^c^	EBV	Selected^d^	SD of ${\mathbf{c}}_{{\mathbf{s}}}$ ^e^
Angler								
*REF*	0.022	0.926	0.527	0.049	0.722	0.211	–	–
*TS*	0.031	0.939	0.565	0.067	0.722	1.184	0.065	0
*minMC.A*	*0.030*	0.827	0.345	0.083	0.570	−0.289	0.106	0.012
*minMC.A.EBV*	*0.030*	0.866	0.404	0.084	0.622	*0.516*	0.091	0.012
*minMC.A.B.EBV*	*0.030*	***0.866***	0.404	0.084	0.622	*0.516*	0.091	0.012
*minMC.A.C.EBV*	*0.030*	0.866	***0.404***	0.084	0.622	*0.516*	0.091	0.012
*minMC.A.D.EBV*	***0.020***	0.903	0.472	*0.046*	0.683	*0.516*	0.342	0.005
Vorderwald								
*REF*	0.030	0.852	0.380	0.072	0.605	0.287	–	–
*TS*	0.043	0.882	0.432	0.093	0.645	1.161	0.106	0
*minMC.A*	*0.035*	0.789	0.300	0.074	0.527	−0.111	0.276	0.011
*minMC.A.EBV*	*0.035*	0.813	0.327	0.075	0.555	*0.550*	0.220	0.010
*minMC.A.B.EBV*	*0.035*	***0.813***	0.327	0.075	0.555	*0.550*	0.220	0.010
*minMC.A.C.EBV*	*0.035*	0.813	***0.327***	0.075	0.555	*0.550*	0.211	0.010
*minMC.A.D.EBV*	***0.031***	0.825	0.342	*0.067*	0.571	*0.550*	0.317	0.008

^a^The name of each optimization scenario consists of a prefix indicating the objective function and a suffix indicating the constraint settings

^b^The parameter used as a constraint is marked in italic in the scenario. Bold italic values show that the actual value obtained does not reach the limit of the corresponding constraint in this scenario (value higher than the lower limit or lower than the upper limit)

^c^Objective function

^d^Proportion of selected sires with non-zero genetic contributions; a ${\text{c}}_{{{\text{s}}_{\text{i}} }}$ value lower than 0.00025 is treated as zero

^e^Standard deviation of the genetic contributions of all male selection candidates

### Maximizing the average EBV

Results for maximizing the average EBV in the progeny generation under various constraints are in Table 7. For both breeds, the *REF* scenario achieved the lowest average EBV in the offspring generation. This value was not zero because male and female selection candidates had different mean EBV. For Angler cattle, scenario *maxEBV.A* achieved a higher EBV (1.226 vs. 1.184) than the TS scenario, although the average kinship ${\text{f}}_{\text{A}}$ was restricted (0.030 vs. 0.031). The average EBV decreased when adding the MC restriction, and ${\text{f}}_{\text{B}}$ and ${\text{f}}_{\text{C}}$ decreased to a level lower than their upper bound settings. Restricting ${\text{f}}_{\text{D}}$ also lowered ${\text{f}}_{\text{A}}$. The EBV dropped to its lowest value of 0.449 when restricting ${\text{f}}_{\text{A}}$, ${\text{f}}_{\text{D}}$ and MC, although this was still around twice that obtained in the *REF* scenario (0.211). Similar results were observed for the Vorderwald cattle population. Scenario *maxEBV.A* achieved a similar EBV as the *TS* scenario (1.164 vs. 1.161) but the average kinship ${\text{f}}_{\text{A}}$ was much lower (0.035 vs. 0.043). When adding restrictions on ${\text{f}}_{\text{D}}$ and MC, the maximum EBV decreased to 0.636, which was more than twice that obtained in the *REF* scenario (0.287).

**Table 7:** Optimization of the genetic contribution when maximizing the breeding value with restricted kinship and/or mean estimated migrant contributions

Scenario^a^	Parameter^b^							
	${\mathbf{f}}_{{\mathbf{A}}}$	${\mathbf{f}}_{{\mathbf{B}}}$	${\mathbf{f}}_{{\mathbf{C}}}$	${\mathbf{f}}_{{\mathbf{D}}}$	MC	EBV^c^	Selected^d^	SD of ${\mathbf{c}}_{{\mathbf{s}}}$ ^e^
Angler								
*REF*	0.022	0.926	0.527	0.049	0.722	0.211	–	–
*TS*	0.031	0.939	0.565	0.067	0.722	1.184	0.065	0
*maxEBV.A*	*0.030*	0.937	0.560	0.082	0.743	1.226	0.085	0.012
*maxEBV.A.MC*	*0.030*	0.901	0.471	0.082	*0.677*	0.979	0.070	0.012
*maxEBV.A.B.MC*	*0.030*	***0.893***	0.454	0.082	***0.664***	0.884	0.075	0.012
*maxEBV.A.C.MC*	*0.030*	0.901	***0.471***	0.082	*0.677*	0.979	0.070	0.012
*maxEBV.A.D.MC*	***0.020***	0.899	0.464	*0.046*	*0.677*	0.449	0.347	0.005
Vorderwald								
*REF*	0.030	0.852	0.380	0.072	0.605	0.287	–	–
*TS*	0.043	0.882	0.432	0.093	0.645	1.161	0.106	0
*maxEBV.A*	*0.035*	0.895	0.456	0.077	0.666	1.164	0.203	0.013
*maxEBV.A.MC*	*0.035*	0.835	0.357	0.079	*0.582*	0.812	0.220	0.011
*maxEBV.A.B.MC*	*0.035*	***0.832***	0.353	0.078	***0.579***	0.787	0.220	0.011
*maxEBV.A.C.MC*	*0.035*	0.835	*0.356*	0.078	***0.581***	0.808	0.220	0.011
*maxEBV.A.D.MC*	***0.031***	0.834	0.354	*0.067*	*0.582*	0.636	0.317	0.008

^a^The name of each optimization scenario consists of a prefix indicating the objective function and a suffix indicating the constraint settings

^b^The parameter used as a constraint is marked in italic in the scenario. Bold italic values show that the actual value obtained does not reach the limit of the corresponding constraint in this scenario (value higher than the lower limit or lower than the upper limit)

^c^Objective function

^d^Proportion of selected sires with non-zero genetic contributions; a ${\text{c}}_{{{\text{s}}_{\text{i}} }}$ value lower than 0.00025 is treated as zero

^e^Standard deviation of the genetic contributions of all male selection candidates

The number of selected sires with non-zero genetic contributions was calculated in each scenario, as well as the standard deviation of the genetic contribution of all male selection candidates. Among all scenarios, TS selected the smallest number of sires. Adding a constraint on ${\text{f}}_{\text{D}}$ resulted in all cases in more selected sires and a lower standard deviation.

## Discussion

For the breeding schemes of the two breeds considered in this study, two conflicts must be addressed: (1) the conflict between increasing genetic gain while managing inbreeding and (2) the conflict between maintaining genetic diversity while controlling loss of genetic uniqueness. The purpose of this study was to determine whether OCS with additional constraints that involve modified kinship matrices and MC was more efficient at conserving genetic diversity and originality while also ensuring genetic improvement than traditional OCS. Using data on German Angler and Vorderwald cattle, various scenarios were compared. Both breeds have been frequently crossed with high-yielding breeds to improve performance. We found that diversity of native alleles decreased more rapidly in Vorderwald cattle than in Angler cattle, whereas MC was higher in Angler cattle. The consequences of the scenarios were similar for both breeds. Compared to traditional OCS, constraining kinship ${\text{f}}_{\text{D}}$ and MC promoted recovery of genetic originality in the breeds and diversity of native alleles but reduced response to selection.

Traditional OCS achieved the highest average EBV in the progeny generation among all scenarios with a restriction on rate of inbreeding, which, in our study, is represented by scenario *maxEBV.A*. Compared to the *TS* scenario, average EBV was higher in the traditional OCS scenario for both breeds, while the average relatedness was lower. Probably, the average EBV in TS was smaller because the TS scenario assumed equal contributions for selected sires, whereas OCS optimizes their contributions. Because MC and EBV were positively correlated, traditional OCS increased the average MC, which is undesirable when the aim is to conserve the genetic originality of local breeds.

### Different kinship estimates

Both ${\text{f}}_{\text{B}}$ and ${\text{f}}_{\text{C}}$ take probabilities of IBD and probabilities of alleles originating from migrant breeds into account, i.e. they account for both level of inbreeding and level of genetic originality. Although theoretically, MC affects ${\text{f}}_{\text{B}}$ more than ${\text{f}}_{\text{C}}$, results from minimizing ${\text{f}}_{\text{B}}$ and ${\text{f}}_{\text{C}}$ were almost identical for the two breeds considered. Wellmann et al. [22] reported a larger difference between these two methods, which is probably because contributions of both sexes were optimized in their work. Minimizing neither ${\text{f}}_{\text{B}}$ nor ${\text{f}}_{\text{C}}$ reduced the kinship at native alleles, ${\text{f}}_{\text{D}}$, thus these two criteria are not an alternative for controlling the kinship at native alleles directly. Results from minimizing ${\text{f}}_{\text{B}}$ and ${\text{f}}_{\text{C}}$ were very similar to the results from minimizing MC. Hence, instead of minimizing or constraining ${\text{f}}_{\text{B}}$ or ${\text{f}}_{\text{C}}$, it is recommended to control MC. To control the diversity at native alleles, ${\text{f}}_{\text{D}}$ must be constrained or minimized directly, although this optimization problem may be not convex. However, because minimizing ${\text{f}}_{\text{D}}$ did not reduce MC, a constraint on MC is needed for all optimizations that involve ${\text{f}}_{\text{D}}$. Minimizing ${\text{f}}_{\text{D}}$ is different from minimizing ${\text{f}}_{\text{A}}$ with an additional constraint on MC because minimizing ${\text{f}}_{\text{A}}$ resulted in a larger ${\text{f}}_{\text{D}}$ than minimizing ${\text{f}}_{\text{D}}$ when MC is constrained to the same level (results not shown). Similarly, when including kinship ${\text{f}}_{\text{D}}$ as an additional constraint in the OCS, the level of kinship ${\text{f}}_{\text{A}}$ decreased in all scenarios. Thus, if ${\text{f}}_{\text{D}}$ is constrained, then MC must be constrained as well and the constraint for ${\text{f}}_{\text{A}}$ can be omitted.

Among all the scenarios, TS used the smallest number of sires and resulted in the highest average genetic contribution of selected sires. Including kinship ${\text{f}}_{\text{D}}$ as an additional constraint in the OCS scenarios resulted in a larger number of selected sires than including ${\text{f}}_{\text{B}}$ or ${\text{f}}_{\text{C}}$. Therefore, including ${\text{f}}_{\text{D}}$ is an efficient method to avoid overuse of sires with high EBV and limits the rate of inbreeding in the long run. Compared with the inclusion of ${\text{f}}_{\text{B}}$ or ${\text{f}}_{\text{C}}$, inclusion of ${\text{f}}_{\text{D}}$ resulted in a lower average EBV in the progeny generation, depending on the constraint level setting. In most cases, OC was negatively correlated with MC and positively correlated with the average EBV, as illustrated in Additional file 2: Table S2, which represents a desirable result for future selection and breeding programs.

Scenarios with optimizations of both male and female contributions were also evaluated (results not shown), using the same calculation methods to obtain the constraint value settings. For all scenarios and both breeds, the constraint settings were stricter than in the scenarios that optimized male contributions. The performance of all scenarios improved when both male and female selection were optimized, which is consistent with Sánchez-Molano et al. [8], who used OCS to improve fitness and productivity traits. To achieve these improvements, however, additional reproductive techniques must be applied due to the limited reproduction rate of female animals.

### Considering the migrant contribution

Previous OCS approaches for maximizing genetic gain while limiting rate of inbreeding did not consider MC. Introgression of migrant breed alleles must be managed to maintain genetic uniqueness and conserve local breeds. As expected, the average EBV obtained with and without MC as a constraint showed that controlling MC restricts increases in genetic gain. Interestingly, kinship at native alleles increased compared to the *REF* scenario when MC was constrained or minimized. Hence, the individuals with the lowest MC may not carry some native alleles that are still present in individuals with higher MC. Thus, in this case, constraining ${\text{f}}_{\text{D}}$ is required to ensure that native genetic diversity is maintained.

However, maximum genetic gains can only be achieved by allowing for the introgression of foreign genetic material. Therefore, the two main purposes in a breeding program, i.e. conserving local breeds and improving genetic gain, are contradictory and must be balanced by the breeding organization. In this study, we set the proportion of breeding progress to be achieved at $\uplambda = \, 0.3$ to determine the constraint level required for achieving optimal solutions for both breeds. Depending on the situation, the breeding organization could select an appropriate value of $\uplambda$ to emphasize conservation of local breeds or genetic improvement, thus facilitating both purposes.

### Future improvements

Because of advances in molecular genetics, genome-wide dense marker genotype data are increasingly available, even for some endangered breeds and have shown promise in capturing genetic variation due to Mendelian sampling [29]. The application of genomic data provides a more accurate method of calculating relationships between individuals compared with the use of estimates from pedigree data [30]. Breeding values estimated by genomic approaches are also more accurate and show more within-family variation compared with breeding values estimated via traditional approaches [31]. Furthermore, compared to the use of pedigree kinship, the use of genomic kinship is substantially more efficient in maintaining genetic diversity when optimizing genetic contributions [8, 12, 16, 32]. Moreover, new methods to estimate kinship at native alleles, i.e. ${\text{f}}_{\text{D}}$, can be developed based on genomic data and the use of genomic data may enable estimation of MC for selection candidates without using pedigree data.

## Conclusions

Maintaining genetic originality is essential for conserving local breeds. It was shown that using an OCS approach as developed in this study can effectively maintain the diversity of native alleles and genetic originality, while ensuring genetic improvement. The most promising approach involved the inclusion of additional constraints for migrant contributions and kinship at native alleles ${\text{f}}_{\text{D}}$. When a constraint for ${\text{f}}_{\text{D}}$ was included, the classical kinship ${\text{f}}_{\text{A}}$ in the offspring was lower than the constraint level, so the constraint on ${\text{f}}_{\text{A}}$ could be removed. More sires were selected when ${\text{f}}_{\text{D}}$ was constrained than when ${\text{f}}_{\text{D}}$ was not constrained and the standard deviation of the contributions was lower, i.e., the optimum contributions of the selected sires were more similar.

## Additional files

**Additional file 1: Table S1.** Threshold settings for all parameters for the Angler and Vorderwald populations.

**Additional file 2: Table S2.** Correlation between OC and EBV and between OC and MC.
