# Supplementary material for: Novel optimum contribution selection methods accounting for conflicting objectives in breeding programs for livestock breeds with historical migration
Source: Genet Sel Evol. 2017 May 12;49:45. doi: 10.1186/s12711-017-0320-7 (PMC5427594; doi:10.1186/s12711-017-0320-7)
Supplement: Supplementary file 1 — Additional file 1: Table S1. Threshold settings for all parameters for the Angler and Vorderwald populations. [file 12711_2017_320_MOESM1_ESM.docx]

|  | **Angler** | | |  | **Vorderwald** | | |
| --- | --- | --- | --- | --- | --- | --- | --- |
|  | $\mathbf{Parameter}_{\mathbf{REF}}$**^1^** | $\mathbf{Limit}$**^2^** | **Constraint** |  | $\mathbf{Parameter}_{\mathbf{REF}}$**^1^** | $\mathbf{Limit}$**^2^** | **Constraint** |
| $f_{A}$ | - | - | 0.030 |  | - | - | 0.035 |
| $f_{B}$ | 0.926 | 0.827 | 0.896^3^ |  | 0.852 | 0.789 | 0.833 |
| $f_{C}$ | 0.527 | 0.345 | 0.472 |  | 0.380 | 0.300 | 0.356 |
| $f_{D}$ | 0.049 | 0.040 | 0.046 |  | 0.072 | 0.057 | 0.067 |
| MC | 0.722 | 0.570 | 0.677 |  | 0.605 | 0.527 | 0.582 |
| EBV | 0.211 | 1.226 | 0.516 |  | 0.287 | 1.164 | 0.550 |

^1^Output obtained from the reference scenario (*REF*)

^2^Upper or lower limit of the parameter in the scenario of the corresponding objective function with the constraint $f_{A}$

^3^ Constraint value for $f_{B}$ is calculated as $ub.f_{B}=\lambda\mathbf{c}_{minfB.A}^{'}\mathbf{f}_{\mathbf{B}}\mathbf{c}_{minfB.A}+(1-\lambda)\mathbf{c}_{\mathrm{REF}}^{'}\mathbf{f}_{\mathbf{B}}\mathbf{c}_{\mathrm{REF}}$ , In this study, we set $\lambda$ to 0.3. Therefore, for Angler cattle,$ub.f_{B}$=0.3*0.827+(1-0.3)*0.926=0.896. The other following constraints settings were similarly calculated except for constraint $f_{A}$.
