# Supplementary material for: Novel optimum contribution selection methods accounting for conflicting objectives in breeding programs for livestock breeds with historical migration
Source: Genet Sel Evol. 2017 May 12;49:45. doi: 10.1186/s12711-017-0320-7 (PMC5427594; doi:10.1186/s12711-017-0320-7)
Supplement: Supplementary file 2 — Additional file 2: Table S2. Correlation between OC and EBV and between OC and MC. [file 12711_2017_320_MOESM2_ESM.docx]

|  | **Angler** | |  | **Vorderwald** | |
| --- | --- | --- | --- | --- | --- |
| **Scenario** | **Cor(OC,EBV)^1^** | **Cor(OC,MC)^2^** |  | **Cor(OC,EBV)^1^** | **Cor(OC,MC)^2^** |
| *minfB.A* | -0.217 | -0.573 |  | -0.323 | -0.521 |
| *minfB.A.MC* | -0.217 | -0.573 |  | -0.323 | -0.521 |
| *minfB.A.EBV.MC* | 0.130 | -0.375 |  | 0.216 | -0.341 |
|  |  |  |  |  |  |
| *minfC.A* | -0.219 | -0.574 |  | -0.325 | -0.526 |
| *minfC.A.MC* | -0.219 | -0.574 |  | -0.325 | -0.526 |
| *minfC.A.EBV.MC* | 0.131 | -0.378 |  | 0.217 | -0.344 |
|  |  |  |  |  |  |
| *minfD.A* | -0.077 | 0.301 |  | 0.276 | 0.310 |
| *minfD.A.MC* | -0.153 | -0.508 |  | -0.188 | -0.255 |
| *minfD.A.EBV.MC* | 0.324 | -0.428 |  | 0.308 | -0.225 |
|  |  |  |  |  |  |
| *minMC.A* | -0.213 | -0.569 |  | -0.322 | -0.518 |
| *minMC.A.EBV* | 0.128 | -0.371 |  | 0.217 | -0.343 |
| *minMC.A.B.EBV* | 0.128 | -0.371 |  | 0.217 | -0.343 |
| *minMC.A.C.EBV* | 0.128 | -0.371 |  | 0.217 | -0.343 |
| *minMC.A.D.EBV* | 0.341 | -0.394 |  | 0.272 | -0.290 |
|  |  |  |  |  |  |
| *maxEBV.A* | 0.427 | 0.076 |  | 0.563 | 0.326 |
| *maxEBV.A.MC* | 0.322 | -0.169 |  | 0.420 | -0.154 |
| *maxEBV.A.B.MC* | 0.285 | -0.219 |  | 0.401 | -0.175 |
| *maxEBV.A.C.MC* | 0.322 | -0.169 |  | 0.419 | -0.158 |
| *maxEBV.A.D.MC* | 0.268 | -0.454 |  | 0.365 | -0.201 |

^1^Correlation between the genetic contributions and estimated breeding values of all male selection candidates in the corresponding scenario

^2^Correlation between the genetic contributions and migrant contributions of all male selection candidates in the corresponding scenario
